# Antileishmanial Activity of Semisynthetic Lupane Triterpenoids Betulin and Betulinic Acid Derivatives: Synergistic Effects with Miltefosine

**DOI:** 10.1371/journal.pone.0089939

**Published:** 2014-03-18

**Authors:** Maria C. Sousa, Raquel Varandas, Rita C. Santos, Manuel Santos-Rosa, Vera Alves, Jorge A. R. Salvador

**Affiliations:** 1 Faculty of Pharmacy, University of Coimbra, Pólo das Ciências da Saúde, Azinhaga de Santa Comba, Coimbra, Portugal; 2 Centre of Pharmaceutical Studies, Faculty of Pharmacy of University of Coimbra (CEF/FFUC), Pólo das Ciências da Saúde, Azinhaga de Santa Comba, Coimbra, Portugal; 3 CNC- Center for Neurosciences and Cell Biology, University of Coimbra, Coimbra, Portugal; 4 Institute of Immunology, Faculty of Medicine, University of Coimbra, Pólo I, Rua Larga, Coimbra, Portugal; University of Bologna & Italian Institute of Technology, Italy

## Abstract

Leishmaniasis is a neglected tropical disease (NTDs), endemic in 88 countries, affecting more than 12 million people. The treatment consists in pentavalent antimony compounds, amphotericin B, pentamidine and miltefosine, among others. However, these current drugs are limited due to their toxicity, development of biological resistance, length of treatment and high cost. Thus, it is important to continue the search for new effective and less toxic treatments.

The anti-*Leishmania* activity of sixteen semisynthetic lupane triterpenoids derivatives of betulin (BT01 to BT09) and betulinic acid (AB10 to AB16) were evaluated. Drug interactions between the active compounds and one current antileishmanial drug, miltefosine, were assessed using the fixed ratio isobologram method. In addition, effects on the cell cycle, apoptosis/necrosis events, morphology and DNA integrity were studied. The derivatives BT06 (3β-Hydroxy-(20R)-lupan-29-oxo-28-yl-1H-imidazole-1-carboxylate) and AB13 (28-(1H-imidazole-1-yl)-3,28-dioxo-lup-1,20(29)-dien-2-yl-1H-imidazole-1-carboxylate) were found to be the most active, with IC_50_ values of 50.8 µM and 25.8 µM, respectively. Interactions between these two compounds and miltefosine were classified as synergistic, with the most effective association being between AB13 and miltefosine, where decreases of IC_50_ values to 6 µM were observed, similar to the miltefosine activity alone. AB13 induced significant morphological changes, while both derivatives produced anti-proliferative activity through cell cycle arrest at the G0/G1 phase. Neither of these derivatives induced significant apoptosis/necrosis, as indicated by phosphatidylserine externalization and DNA fragmentation assays. In addition, neither of the derivatives induced death in macrophage cell lines. Thus, they do not present any potential risk of toxicity for the host cells.

This study has identified the betulin derivative BT06 and the betulinic acid derivative AB13 as promising molecules in the development of new alternative therapies for leishmaniasis, including those involving combined-therapy with miltefosine.

## Introduction

Over one billion people are infected by one or more neglected tropical diseases (NTDs). These diseases comprise a group of parasitic, viral and bacterial infections that affect some of the poorest and most marginalized populations globally. Leishmaniasis is one such NTD that is endemic in 88 countries, affecting more than 12 million people and threatening 350 million people worldwide. The disease is associated with an incidence of 1.5 to 2 million cases per year, and an annual mortality rate of over 59,000 deaths [1]. It is caused by the *Leishmania* species, and the disease is broadly classified into three different clinical forms: visceral leishmaniasis (VL), cutaneous leishmaniasis (CL) and mucocutaneous leishmaniasis (MCL), which differ in the pattern and clinical manifestations of infection. VL can be fatal if left untreated, CL is localized and frequently self-heals within 3–18 months, while MCL leaves disfiguring scars. The parasite growth occurs through different morphological stages: the flagellated promastigotes develop in the gut of the phlebotomine sandfly female (vector), while the non-flagellated amastigotes develop in mammalian host macrophages.

Currently, the treatment consists of chemotherapeutic agents, such as the pentavalent antimony compounds (sodium stibogluconate or meglumine antimoniate), polyene amphotericin B (as the deoxycholate salt or a liposomal formulation, AmBisome), the alkylphosphocholine miltefosine, aminoglycoside paromomycin and pentamidine [2]. All of these are limited due to their high toxicity, life-threatening side-effects, cost, length of treatment and emergence of resistance [1], [3]. Because of the limited viable treatment options, with few alternatives available in the pipeline, it is important to continue the search for new effective chemotherapeutics and less toxic treatments.

The development of drug resistance is associated with monotherapy regimes [3], [4]. Combination therapy is one interesting approach to decrease this development, to reduce the duration and cost of the treatment, and to increase the lifetime of old and new drugs [5], [6]. Several combined treatments for visceral leishmaniasis have been tested with positive results, leading to a reduction of adverse symptoms and a shorter duration of therapy [7].

Recent pharmacological studies elicited interest in several molecules with activities that trigger apoptotic death in cancerous cells as potential antiparasitic agents [8]. In trypanosomatids, features suggesting programmed cell death (PCA), commonly named as apoptosis, have been reported in response to a wide range of stimuli such as heat shock, reactive oxygen species, antiparasitic drugs, prostaglandins, and antimicrobial peptides [9]–[11]. There are several reports showing that *Leishmania* apoptosis occur in response to antileishmanial drugs. The treatment with the pentavalent antimony Sb(V) shows a significant induction of caspase-like activity resulting in DNA fragmentation [12]. Inhibitors of respiratory chain complexes were able to induce apoptotic cell death on the blood stream form of *L.* donovani [13]. Topoisomerase I poison camptothecin, promotes protein-DNA cleavable complex formation leading to apoptosis-like cell death in *Leishmania donovani* [14], [15]. Miltefosine, the latest antileishmanial drug introduced in the market and the first effective oral treatment of VL, was initially developed as an anticancer drug [16] and induce apoptosis-like cell death in *Leishmania donovani* [17]. All these findings point the importance of testing known anticancer agents against *Leishmania* due of its potential to inducing parasite death.

Triterpenoids in general, and particularly betulin and its derivative betulinic acid, show antitumor [18]–[20], anti-inflammatory [21], [22], antiviral [19], [23], [24], antibacterial and antimalarial activity [25], [26]. There are few studies in literature reporting the activity of betulin and betulinic acid derivatives in *Leishmania*. Alakurtti and collaborators [27] determined the activity of heterocyclic betulin derivatives on *Leishmania donovani* amastigotes and Dominguez-Carmona and collaborators [28] found activity against promastigotes of *L. amazonensis* of betulinic acid acetate and of betulinic acid methyl ester.

Previously, a series of new imidazole carboxylic esters (carbamates) and N-acylimidazole derivatives of betulin and betulinc acid have been synthesized and were showed to have cytotoxicity activity against human cancer cell lines HepG2, Jurkat and HeLa [29]. This cytotoxicity was related with apoptosis mechanisms associated with caspases signalling and DNA topoisomerases inhibition [30], [31]. These results prompt us to evaluate their potential activity on *Leishmania*. We studied the susceptibility of *Leishmania infantum* to sixteen betulin and betulinic acid derivatives, together with the interactions between the most active compounds and miltefosine. Additionally, we have undertaken other assays to demonstrate the safety of the triterpenoid compounds for mammalian cells, and to elucidate the mechanisms of action involved in the leishmanicidal activity.

## Materials and Methods

### Chemicals and reagents

RPMI-1640 Medium, Phosphate Buffered Saline (PBS), 3-(4,5-dimethylthiazol-2-yl)-2,5-diphenyltetrazolium bromide (MTT), dimethylsulfoxide (DMSO) were obtained from Sigma–Aldrich Co. Fetal bovine serum (FBS) was purchased from Gibco-Invitrogen.

### Drugs and inhibitors

**Figure 1:**
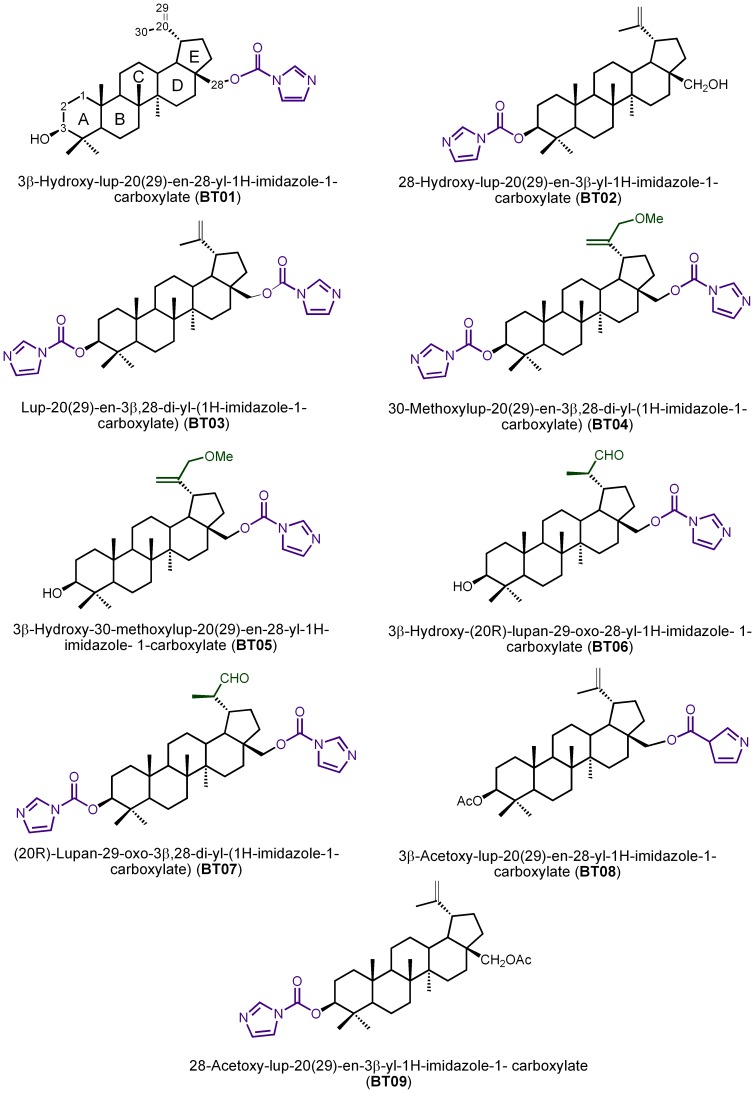
Structures of betulin derivatives and IUPAC nomenclature.

**Figure 2:**
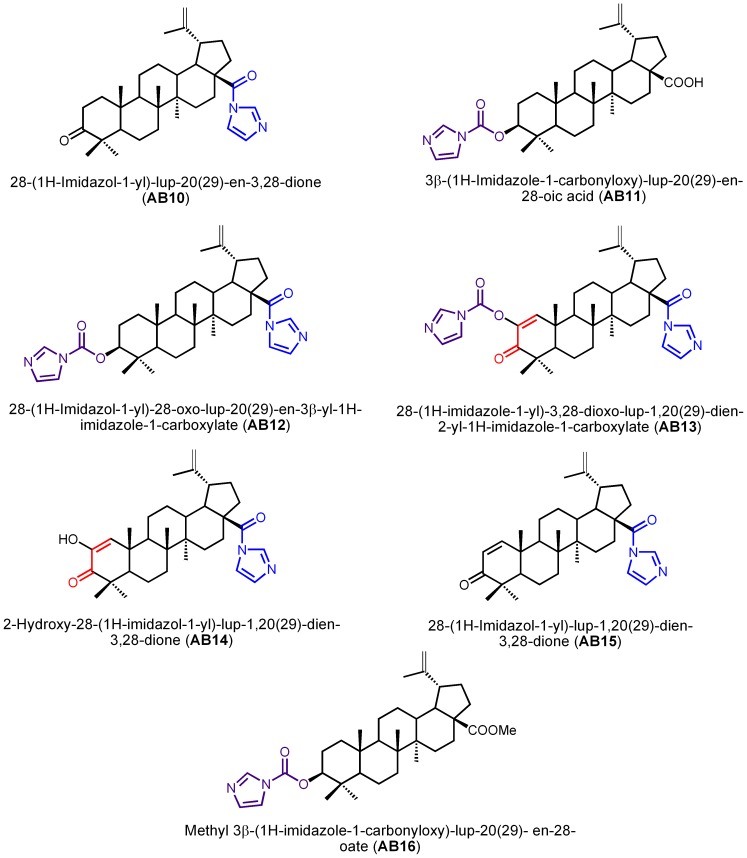
Structures of betulinic acid derivatives and IUPAC nomenclature.

Miltefosine was obtained from Sigma Chemical Co.(St. Louis,USA). The nine derivatives of betulin (Figure 1) and the seven betulinic acid derivatives (Figure 2) used in this study were previously synthesized as part of a large library of compounds for potential antitumor evaluation [29]. The stock solutions (10 mM) derivatives of betulin and betulinic acid were prepared in dimethylsulfoxide (DMSO) and stored at −20°C. A stock solution of miltefosine (10 mM) was prepared in deionizer water and stored at 4°C. Final DMSO concentration (1%) had no effect on parasite clearance.

### Parasites and cultures

Promastigote forms of *Leishmania infantum* Nicolle (zymodeme MON-1) were maintained at 26°C with weekly transfers in HEPES (25 mM)-buffered RPMI 1640 medium enriched with 10% inactivated fetal bovine serum (FBS). Log phase promastigotes were used to study the effects of the betulin and betulinic acid derivatives, miltefosine and drug combinations.

### Viability assay

In drug susceptibility assays, stock solutions of betulin and betulinic acid derivatives were diluted in culture medium (RPMI 1640) in order to get a range of concentrations from 1 to 100 µM. Log phase promastigotes of *L. infantum* (2×10^6^ cells.ml^−1^) were incubated at 26°C for 24 h in HEPES (25 mM)-buffered RPMI 1640 medium enriched with 10% inactivated FBS in the presence of increasing concentrations of the derivatives or different concentration of DMSO (maximum of 1% as controls).

The effect on the cells viability was tested by MTT (3-[4, 5-methylthiazol-2-yl]-2, 5-diphenyltetrazolium bromide) colorimetric method, based on the reduction of the tetrazolium dye to insoluble formazan by the mitochondrial enzymes [32]. Briefly, 25 µl of MTT (5 mg.ml^−1^) was added to each well, incubated for 2 h at 37°C and centrifuged at 3000 rpm for 5 min. The supernatant was removed, the cells were washed in PBS, and the precipitated formazan was dissolved in DMSO (250 µl). Cell viability was measured by absorbance at 530 nm on an ELISA plate reader (Synergy HT, Bio-TEK), and calculated using the following formula: [(L2/L1)×100], where L1 is the absorbance of control cells and L2 is the absorbance of treated cells. Three separate experiments were performed for each drug and the concentration that inhibited viability by 50% (IC_50_) was determined through dose-response regression analysis, plotted by GraphPad Prism 5.

### Isobologram construction and classification of the nature of drug interaction

For the most active betulin and betulinic acid derivatives, combinations were made with them at growing concentrations and miltefosine at fixed concentrations and cells viability was evaluated by MTT as described above.

Classical isobolograms were constructed by plotting drugs concentrations (alone and in combination) that inhibits 50% trophozoites viability, as previously described [33]. The isobologram analysis evaluates the nature of interaction of two drugs, *i.e.*, drug A and drug B. First, the concentrations of drugs A and B required to produce a defined single-agent effect (*e.g.*, IC50), when used as single agents, are placed on the *x* and *y* axes in a two-coordinate plot, corresponding to (CA, 0) and (0, CB), respectively. The line connecting these two points is the line of additivity. Second, the concentrations of the two drugs used in combination to provide the same effect, denoted as (cA, cB), are placed in the same plot. Synergy, additivity, or antagonism are indicated when (cA, cB) is located below, on, or above the line, respectively.

### Morphological studies

*L. infantum* promastigotes were exposed to the most active betulin and betulinic acid derivatives and the morphological alterations were investigated by optical microscopy using direct examination of live microorganism (hanging drop) and after Giemsa stain. Briefly, exponentially grown of *L. infantum* promastigote (2×10^6^ cells.ml^−1^) were treated with BT06 and AB13 at IC_50_ concentrations for 2 h, 4 h, 6 h and 24 h at 26°C. After incubation, cells were pelleted by centrifugation at 3000 rpm for 5 min and the supernatant was discarded by aspiration. The cell pellet was suspended in fresh medium and approximately 10 µL was placed on a Koch slide and directly observed under the optical microscope phase contrast (Eclipse E400, Nikon coupled with a digital camera 165 DN100 Nikon). In addition, a smear was made which was submitted to Giemsa stain. The smear was fixed with methanol for 5 min, stained with aqueous solution of Giemsa (1/10, v/v) for 10 min at room temperature and finally washed with water and air dried. The stained smear was observed under the microscope with a 100× lens (Eclipse E400, coupled with Nikon digital camera, Nikon DN100 165).

### Cell cycle analysis

For analysis of DNA content, exponentially grown of *L. infantum* (2×10^6^. cells ml^−1^) were treated with BT06 and AB13 at IC_50_ concentrations for 2 h, 4 h, 6 h and 24 h at 26°C. At each time point, cells were fixed in 200 µl of 70% ethanol for 30 min. at 4°C. After washing cells with 2 mL of PBS, enriched with 2% of bovine serum albumin (BSA), the pellets were suspended in 0.5 mL of PI solution (PI/Rnase, Immunostep) and incubated for 15 minutes at 37°C [34]. Cells were then analyzed by flow cytometry (FacsCalibur-Beckton-Dickinson). Results were treated using ModFit LT V 2.0 programme.

### Phosphatidylserine externalization

Double staining for annexin V-FITC and propidium iodide (PI) was performed as described previously [35]. Briefly, *L. infantum* promastigotes (2×10^6^ cells.ml^−1^) were exposed to BT06 and AB13 at IC_50_ concentrations for 2 h, 4 h, 6 h, and 24 h at 26°C. Cells were then washed with PBS and ressuspendedn in binding buffer (10 mM HEPES–NaOH, pH 7.4, 140 NaC1, 2.5 mM CaCI2). To 100 µl of this suspension were added 5 µl of Annexin VFITC and 5 µl of PI (AnnexinV-FITC Apoptosis detection Kit, Immmunostep). After 15 min incubation in the dark at room temperature, it was added 400 µl binding buffer and cells were analyzed by flow cytometry (FacsCalibur–Beckton–Dickinson). Data analysis was carried out using the program Paint-a-gate, and values are expressed as a percentage of positive cells for a given marker, relatively to the number of cells analyzed.

### DNA fragmentation assay

Promastigotes of *L. infantum* (2×10^6^ cells.ml^−1^) were exposed to IC_50_ concentration of the most active betulin and betulinic acid derivatives or to dissolution vehicle (DMSO), and incubated at 26°C for 24 hours. The *Leishmania* DNA extraction was carried out according to the procedure in DNeasy Blood & Tissue (Qiagen). DNA integrity analysis was done by electrophoresis, running DNA through an EtBr-treated agarose gel and visualizing it with UV light.

### Mammalian cell cytotoxicity

For cytotoxicity assays on mammalian cells, log phase of macrophages (RAW 264.7) were trypsinized and incubated at 37°C in 24-well tissue culture plates in Dulbecco's Modified Eagle Medium (DMEM), enriched with Glutamax and supplemented with 10% fetal bovine serum (FBS), under microaerophilic condition. As soon as the monolayers reached confluence, the medium was removed and the cells were incubated at 37°C for 24 h with fresh medium plus the betulin or betulinic acid derivatives at IC_50_ concentrations. After incubation, control and treated cells were washed with PBS, pH 7.2., and 450 µl of PBS and 50 µl MTT solution (5 mg.ml^−1^) were added to each well and incubated at 37°C for 1 h. The cells were then washed with PBS, 500 µl DMSO was added to the wells and absorbance was measured at 530 nm on an ELISA plate reader (Synergy HT, Bio-TEK). The percentage of viable cells was determined as described on viability assay.

### Statistical analysis

All experiments were performed in triplicate. The mean and standard deviation (SEM) of three independent assays were determined and statistical analysis between mean values was done by ANOVA test, with a Dunnett's post-test. The significance level was *p<0.05, **p<0.01 and ***p<0.001.

## Results and Discussion

**Figure 3:**
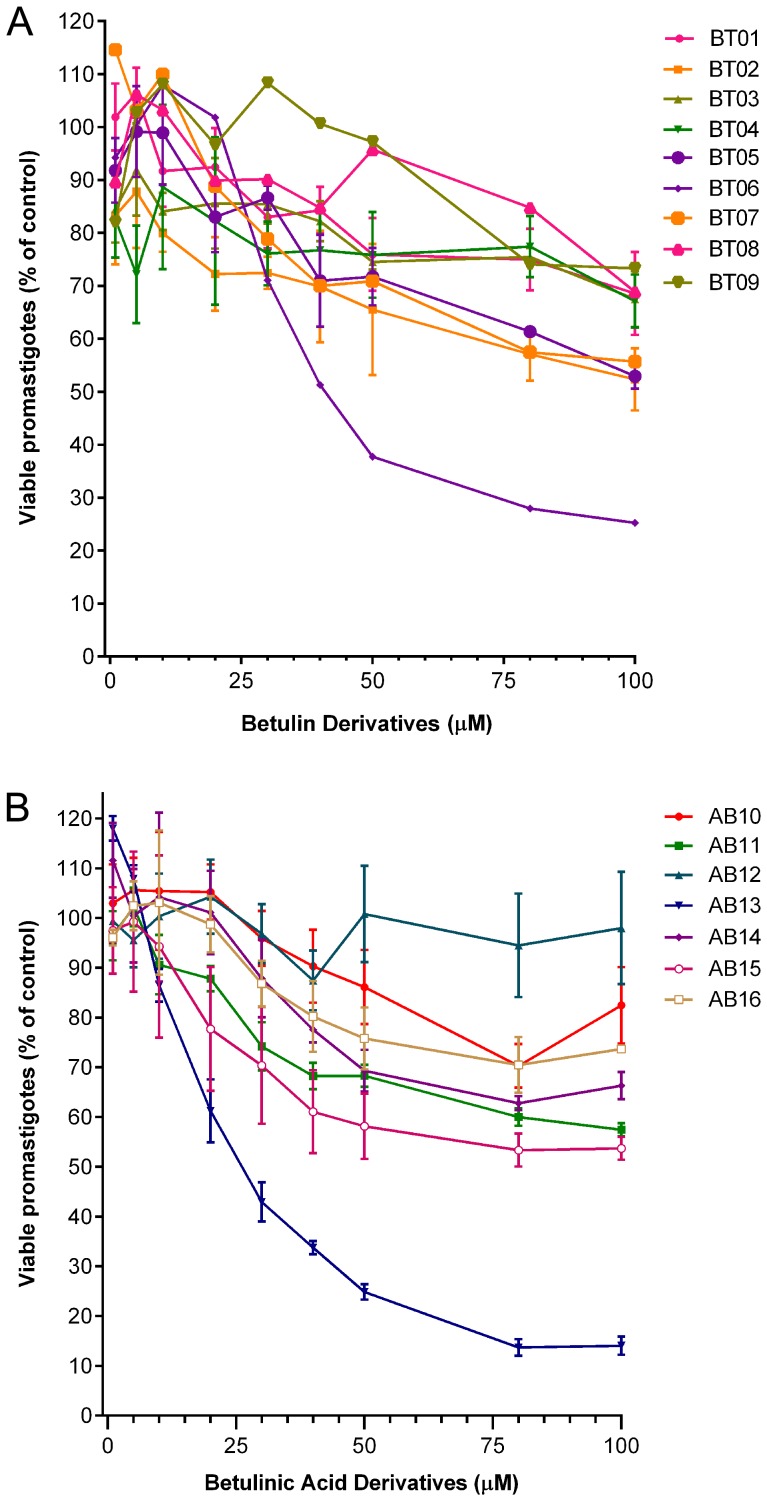
Effects of betulin (A) and betulinic acid (B) derivatives on *Leishmania infantum* promastigotes viability. Cultures of log-phase promastigotes (2×10^6^ cells.ml^−1^) were incubated at 26°C for 24 h at different drug concentrations. Values are expressed as means and SEM.

This study considers the biological potential of the betulin and betulinic acid derivatives, and is aimed at the evaluation of the anti-*Leishmania* activity of 16 synthetic derivatives of betulin (BT01 to BT09) and betulinic acid (AB10 to AB16). This is followed by a study of the association of the most active derivatives with miltefosine, a drug used in the treatment of leishmaniasis. The activity of the derivatives on *L. infantum* was assessed by cell viability studies. At the highest concentrations, all nine betulin derivatives inhibit promastigote viability (Figure 3A). Generally, the results showed that modifications at C-20 (compounds BT06 and BT07, Figure 1) and at C-30 (compound BT05, Figure 1) have a positive impact on the anti-leishmanial activity of betulin derivatives. The introduction of a carbamate moiety at C-28 in the C-3 substituted derivative BT02 (Figure 1) afforded compound BT03 with lower anti-leishmanial activity (Figure 3A). The betulin derivative BT06 (3β-Hydroxy-(20R)-lupan-29-oxo-28-yl-1H-imidazole-1-carboxylate) was found to be the most effective, with an IC_50_ of 50.8 µM. The other derivatives did not inhibit the growth of *L. infantum* by 50% at the highest concentration tested (100 µM).

Of the seven betulinic acid derivatives (Figure 3B), the most active compound was AB13 (28-(1H-imidazole-1-yl)-3,28-dioxo-lup-1,20(29)-dien-2-yl-1H-imidazole-1-carboxylate), with an IC_50_ of 25.8 µM. The remaining betulinic acid derivatives were not able to inhibit viability of *L. infantum* by 50% at the highest concentrations tested (100 µM), however, compounds AB14 with an α,β-unsaturated keto group at ring A (Figure 2), AB15 and AB11 with a keto group and carbamate moiety at C-3 (Figure 2), respectively, showed moderate anti-leishmanial activity. The introduction of the imidazole and methyl esther at C-28 in compounds AB12 and AB16 (Figure 2) respectively, reduce the anti-leishmanial activity (Figure 3B), when compared with derivative AB11 bearing a free carboxylic acid at C-28 position (Figure 2).

The best activity of AB13 compound, is consistent with published results that report a higher biological activity of betulinic acid derivatives compared with betulin ones [19], [31]. The superior anti-*Leishmania* activity of the AB13 derivative could be associated with the higher capacity as a Michael acceptor of this derivative. Considering the Michael addition reaction and according to Sporn and collaborators [36], one possible biological mechanism of action of AB13 may be related to the nucleophilic attack from thiol groups of reduced glutathione or other physiological nucleophiles, including amine nucleophiles, to the α-β-unsaturated-keto group (C1 in ring A) (Figure 2).

The interpretation of results and comparisons between drug activity studies need to take into account the *Leishmania* species and the parasite model cells, i.e., promastigotes or amastigotes. There are only a few reports on the activity of betulin and betulinic acid derivatives in *Leishmania donovani* amastigotes (IC_50_ values of 8.9 to 30 µM) [27] and on promastigotes of *L. amazonensis* (IC_50_ of 44.9 µM to 69.9 µM) [28]. Comparing these with the present data, we observe that BT06 and AB13 derivatives show antileishmanial activity that is similar or higher than that described for the other triterpenoids. In addition, as well as being active against promastigote cells, BT06 and AB13 are expected to exhibit stronger activity on amastigote forms, as tends to be found among natural extracts and synthetic drugs [37]–[41].

It is important in the discovery of new drugs that a molecule with anti-*Leishmania* activity does not impart significant toxicity to the host cells. In this study, we have evaluated the toxicity of the most active derivatives in a macrophage cell line (RAW 264.7). We found that BT06 and AB13 derivatives, at IC_50_ concentrations, were not cytotoxic toward the macrophage cell line, suggesting that they are safe for mammalian cells. Therefore, the two triterpenoides are promising compounds for the discovery of new drugs against *Leishmania* infections.

**Figure 4:**
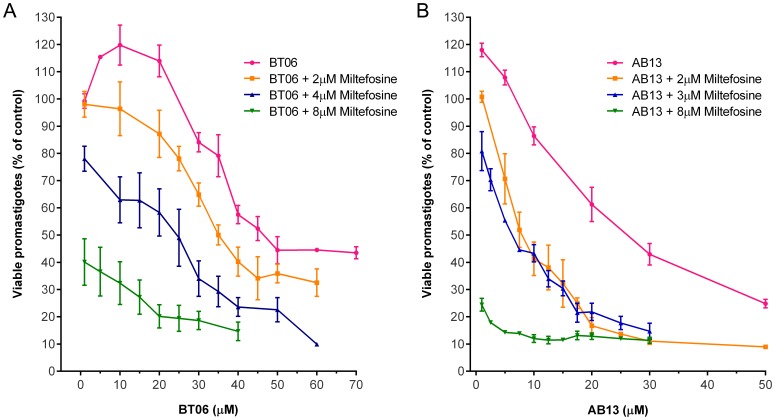
Effects of combinations of betulin and betulinic acid derivatives with miltefosine on *Leishmania infantum* promastigotes viability. (A) Betulin derivative BT06; (B) Betulinic acid derivative AB13. Values are expressed as means and SEM. Cultures of log-phase promastigotes (2×10^6^ cells.ml^−1^) were incubated at 26°C for 24 h at different compound concentrations.

For each of the two most active derivatives (BT06 and AB13), three different combinations at fixed concentrations of miltefosine (2, 4 and 8 µM) were tested. Dose-response curves showed that the combinations of BT06/miltefosine and AB13/miltefosine were more effective at reducing the viability of promastigotes relative to the derivatives alone (Figure 4). The IC_50_ values obtained for all the tested combinations were less than the values obtained for the pure derivatives (Table 1). The combination of the betulin derivative BT06 with miltefosine at 2 µM and 4 µM induced decreases of the IC_50_ value from 50,8 µM to 30,1 µM and 25,9 µM, respectively. The combination of the betulinic acid derivative AB13 with miltefosine at 2 µM and 4 µM induced decreases of the IC_50_ value from 25,8 µM to 7,6 µM and 6,0 µM, respectively.

**Table 1:** Inhibitory concentrations at 50% (IC_50_) of the association between betulin and acid betulinic derivatives (BT06, AB13) with miltefosine on *Leishmania infantum* promastigotes.

Drug combination		IC_50_ µM (CI)*	R^2^
BT06		50.8 (46,9–55.0)	0.85
	+miltefosine 2 µM	30.1 (27,2–33.4)	0.94
	+miltefosine 4 µM	25.9 (20,1–33.3)	0.81
AB13		25.8 (23,3–28.7)	0.92
	+miltefosine 2 µM	7.6 (5,8–10.0)	0.87
	+miltefosine 4 µM	6.0 (5,3–6.7)	0.93
Miltefosine		7.6 (6,6–8.8)	0.93

* CI, confidence interval at 95%.

**Figure 5:**
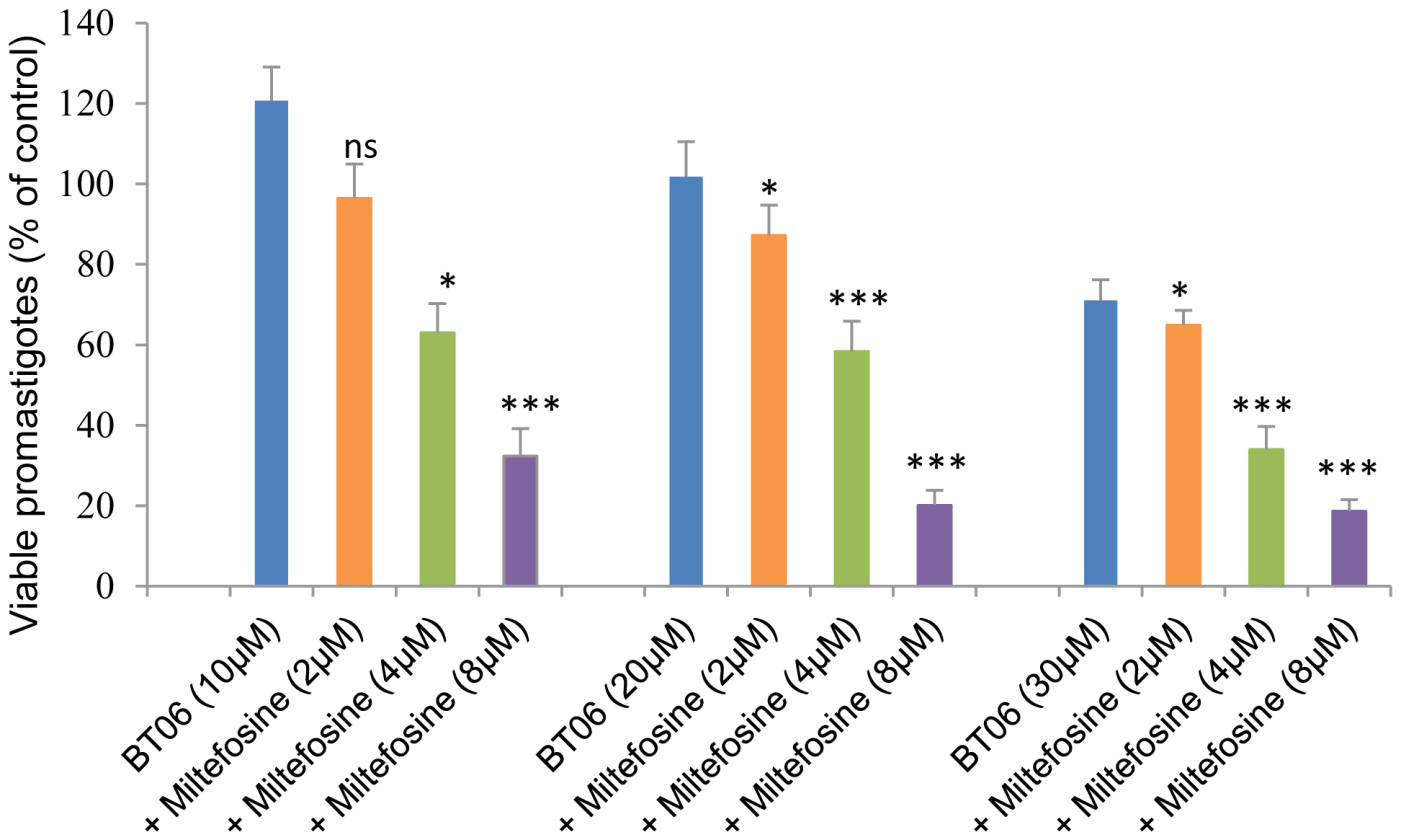
Comparison of the effects of combinations between BT06 and miltefosine with the effects of BT06 alone. Cultures of log-phase promastigotes (2×10^6^ cells.ml^−1^) were incubated at 26°C for 24 h. Significance level of *p<0.05, **p<0.01 and ***p<0.001.

**Figure 6:**
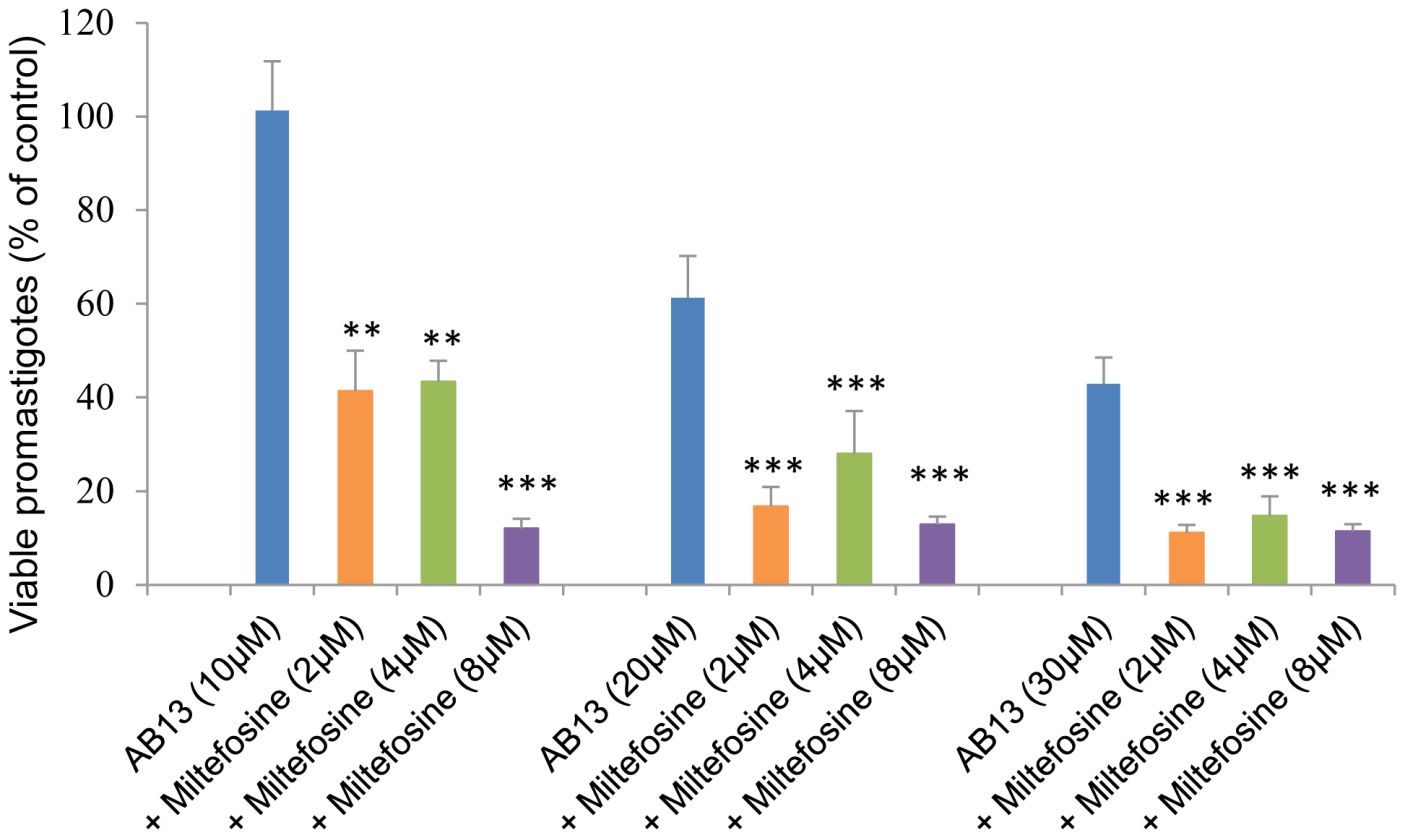
Comparison of the effects of combinations between AB13 and miltefosine with the effects of AB13 alone. Cultures of log-phase promastigotes (2×10^6^ cells.ml^−1^) were incubated at 26°C for 24 h. Significance level of *p<0.05, **p<0.01 and ***p<0.001.

**Figure 7:**
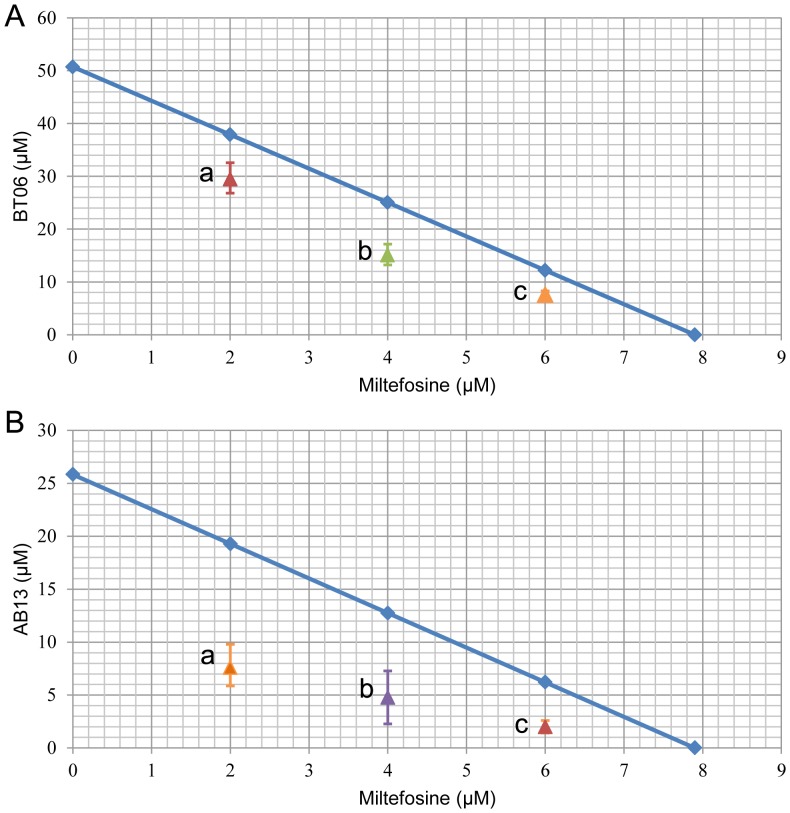
Isobologram analysis for the combinations between BT06 and miltefosine (A) and AB13 and miltefosine (B). The line indicates synergy, additivity or antagonism when the points are located below, on or above the line, respectively. (a) derivative and 2 µM miltefosine; (b) derivative and 4 µM miltefosine; (c) derivative and 6 µM miltefosine.

The statistical differences between the effects of the combinations of the derivatives BT06 and AB13 with miltefosine and the effects of the derivatives alone were analyzed. The combinations between BT06 and miltefosine (Figure 5) induced a significant reduction (p<0.05, p<0.01 and p<0.001) on promastigote viability relative to the BT06 activity, except for the combination BT06 10 µM with miltefosine 2 µM. The association AB13 and miltefosine is most effective, with high statistical significance (p<0.01 and p<0.001) in all combinations when compared with the effects of the derivative alone (Figure 6). Isobolographic analysis showed that all the interactions of AB13 and BT06 with miltefosine were synergistic, with experimental values (a, b, c) below the theoretical IC_50_ values (line of additivity) (Figure 7). The advantages of combination therapy include increased effectiveness of the drug, reduced dosage, decreased toxicity and a delay or prevention of the onset of drug resistance [3]. The derivatives show a synergistic association with miltefosine, therefore they could be promising molecules for the development of new combination therapies for leishmaniasis.

**Figure 8:**
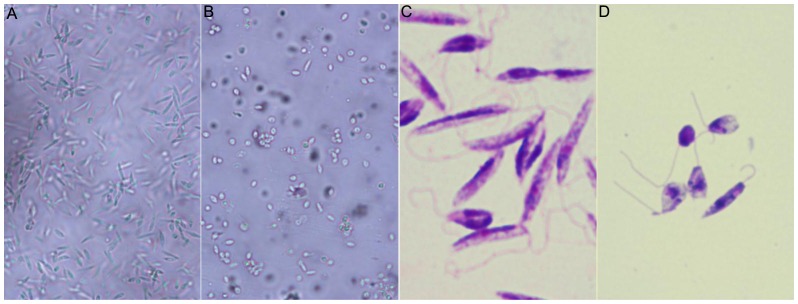
Optical microscopy observation of *Leishmania infantum* promastigotes in the absence and presence of the betulinic acid derivative AB13. Hanging dropin phase contrast (A, B, magnification 200×) and Giemsa staining (C, D, magnification 1000×). Control cells incubated with DMSO at 26°C for 24 h (A, C). Cells exposed to the AB13 derivative at 26°C for 24 h (B, D).

In this work, we have also looked at the mechanisms of action responsible for the biological activity in *Leishmania* of the most active betulin and betulinic acid derivatives (BT06 and AB13). The effects on morphology, apoptosis/necrosis events, DNA integrity and cell cycle were studied. The morphological modifications were evaluated by optical microscopy using direct examination of live microorganism (hanging drop) and after Giemsa stain. Control cells were very mobile with the characteristic fusiform shape (Figure 8A), while after Giemsa staining it was possible to observe the characteristic shape, long flagellum emerging from the anterior region of the parasite, nucleus and kinetoplast, posterior to the nucleus (Figure 8C). With the BT06 derivative, no significant morphologic alterations were observed (not shown). However, the promastigotes exposed to AB13 suffered significant morphological changes (Figure 8 B,D) compared with the control. These cells showed a marked decrease in mobility, and it was possible to observe changes in the size and shape of the promastigotes, which become smaller and rounder (Figure 8B). The Giemsa stained promastigotes also show alterations in flagellum size (Figure 8D). These effects suggest that the leishmanicidal activity of the AB13 derivative may be associated with changes in the cytoskeletal organization and function, and modification of the activity of mitochondrial bioenergetics.

Cell death by apoptosis/necrosis was assessed by flow cytometry using Annexin V/PI labeling and the study of the DNA integrity. During early apoptosis, phosfatidilserine (PS) is translocated from the cytoplasmic face of the plasma membrane to the external face, which can be detected using Annexin V. To distinguish apoptotic cell death from necrotic cell death, cells were counterstained with PI, a non-permeable stain with an affinity for nucleic acids, as it selectively enters necrotic cells. Therefore, co-staining of annexin V and PI can differentiate between cells undergoing early apoptosis (annexin V^+^/PI^−^), necrosis or late apoptosis (PI^+^/annexin V^−^) and live cells (PI^−^/annexin V^−^). The *Leishmania* promastigote cells treated during 24 hours with BT06 and AB13 did not show significant differences in annexine V and PI staining compared with the respective controls (Table 2).

**Table 2:** Flow cytometry analysis of *Leishmania infantum* promastigotes treated with betulin and betulinic acid derivatives (BT06, AB13) showing the percentage of propidium iodide (PI) and annexin-V positive cells.

	*Leishmania infantum* intracellular entities (% of cells)											
	Annexin-V				PI				Annexine/PI			
	2 h	4 h	6 h	24 h	2 h	4 h	6 h	24 h	2 h	4 h	6 h	24 h
**Control** (BT06)	3.8	2	1.6	1.5	0.6	1.2	0.7	0.9	0.7	0.4	0.4	0.6
**BT06**	1.2	2.5	2.6	1.5	0.5	1.4	0.9	0.5	0.5	0.9	0.6	0.2
**Control** (AB13)	18.8	6.3	3.3	2.4	0.4	0.3	0	0.4	3.7	1.5	0.4	0.7
**AB13**	17.7	3.4	4.5	2.6	0.1	0	0.2	0	1.9	0.1	0.9	0.6

**Figure 9:**
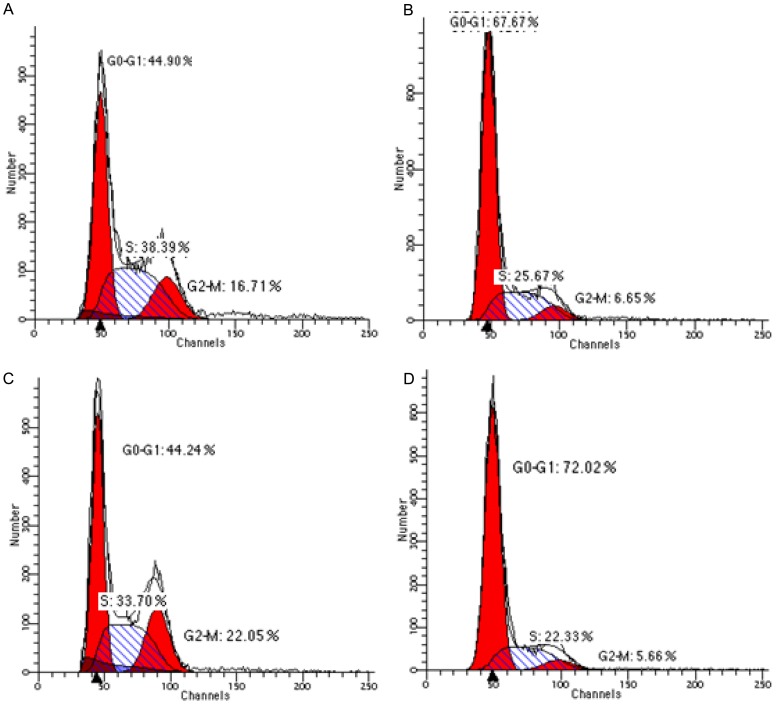
Representative cell cycle histograms of *Leishmania infantum*. Promastigotes were incubated at 26°C for 24 h in the absence (A and C) or presence of betulin derivative BT06 (B) and betulinic acid derivative AB13 (D) at IC_50_ concentrations. Propidium iodide staining was performed and samples were analyzed by flow cytometry.

The cell cycle analysis was performed by flow cytometry after PI staining of the parasites incubated with betulin derivative BT06 and betulinic acid derivative AB13 for 24 h at IC_50_ concentrations (Table 3). Figure 9 shows the distribution of cell DNA trough cell cycle of parasites in the absence and presence of the BT06 and AB13. After 24 h of incubation, the majority of treated parasite cells were on G0/G1 phase of cell cycle (BT06, 67.7%; AB13, 81.2%), which is the opposite of what occurs in non-treated cells (44.2% and 48%, respectively). Both derivatives promoted retention of *L. infantum* promastigotes in the G0/G1 phase of the cell cycle, suggesting an arrest at this stage of the cycle. We observed a marked reduction in DNA replication and mitosis, with a decrease in the number of cells in G2/M phase and S phase mainly with the derivative AB13. Inhibition of *L. infantum* promastigote proliferation seems to represent a major mechanism of activity of derivatives BT06 and AB13.

**Table 3:** Effects of betulin and betulinic acid derivatives on cellular cycle of *Leishmania infantum* promastigotes.

	*Leishmania infantum* intracellular entities (% of cells)											
	Phase G0/G1				Phase S				Phase G2/M			
	2 h	4 h	6 h	24 h	2 h	4 h	6 h	24 h	2 h	4 h	6 h	24 h
**Control** (BT06)	83.6	90.7	72	44.2	16.5	9.9	28	33.7	0	0	0	22.1
**BT06**	92.9	91.8	72.5	67.7	7.1	6.8	28	25.7	0	0	0	6.7
**Control** (AB13)	75.9	75.3	75.5	48	24.1	24.5	24.5	30	0	0	0	22
**AB13**	70.6	75.8	60.9	81.2	29.2	24.2	39.1	18.8	0	0	0	0

It was reported that dihydrobetulinic acid (DHBA), a derivative of betulinic acid, is an excellent inhibitor of *Leishmania* DNA topoisomerase I and II and induces apoptosis in *L. donovani* [42]. More recently, it was shown that three betulin derivatives (disuccinyl betulin, diglutaryl dihydrobetulin, and disuccinyl dihydrobetulin) inhibit both growth of *Leishmania donovani*, and relaxation activity of the enzyme type IB topoisomerase of the parasite (topoisomerase I; LdTOP1LS) [43]. Considering these potential therapeutic targets in *Leishmania* and based on our results, we suggest that *Leishmania* DNA topoisomerases could be a potential target for BT06 and AB13 derivatives. This hypothesis is also supported in the previous antiproliferative activity of AB13 and BT06 derivatives on cancer cell lines and on their inhibitory proprieties on DNA topoisomerase [29]–[31].

In conclusion, the betulin derivative BT06 and betulinic acid derivative AB13 are promising lead compounds which can be used in the discovery of new therapies for *Leishmania* infections, including multidrug treatment schedules with miltefosine.
